# Immediate and longer-term changes in mental health of children with parent–child separation experiences during the COVID-19 pandemic

**DOI:** 10.1186/s13034-023-00659-y

**Published:** 2023-10-04

**Authors:** Peipei Wu, Shihong Wang, Xudong Zhao, Jiao Fang, Fangbiao Tao, Puyu Su, Yuhui Wan, Ying Sun

**Affiliations:** 1 Department of Maternal, Child & Adolescent Health, School of Public Health, Anhui Medical University, 81th Meishan Road, Box 230032, Hefei, Anhui Province China; 2 Key Laboratory of Population Health Across Life Cycle (Anhui Medical University), Ministry of Education of the People’s Republic of China, No 81 Meishan Road, Hefei, 230032 Anhui China; 3 Anhui Provincial Key Laboratory of Population Health & Aristogenics, Hefei, Anhui Province China

**Keywords:** Mental health, The COVID-19 pandemic, Caregiver buffering

## Abstract

**Background:**

The psychological impact of the COVID-19 pandemic has been understudied among vulnerable populations. This study aimed to examine the immediate and longer-term changes in the mental health of children with parent–child separation experiences during the COVID-19 pandemic, and identify potential buffering opportunities for mental health.

**Methods:**

This longitudinal cohort study used data from 723 rural Chinese children who provided data before (Oct. 2019) the COVID-19 pandemic and during the following 2 years. Changes in the probability of depressive symptoms, anxiety symptoms, non-suicide self-injurious (NSSI), suicidal ideation, suicide plan, and suicide attempt were tested across four waves using generalized estimating models (GEE).

**Results:**

Compared with children who never experienced parent–child separation, children persistently separated from parents since birth experienced greater deterioration in all mental health in the 2-year follow-up (average change: depressive symptoms: *β* = 0.59, 95% *CI* [0.26, 0.93]; anxiety symptoms: *β* = 0.45, 95% *CI* [0.10, 0.81]; NSSI: *β* = 0.66, 95% *CI* [0.31, 1.01]; suicide ideation: *β* = 0.67, 95% *CI* [0.38, 0.96]; suicide plan: *β* = 0.77, 95% *CI* [0.38, 1.15]; suicide attempt: β = 1.12, 95% *CI* [0.63, 1.62]). However, children with childhood separation from their parents but reunited with them during the transition to adolescence showed similar even lower changes to counterparts who never experienced parent–child separation (all *ps* > 0.05).

**Conclusion:**

These results indicating improvements in supportiveness of the caregiving environment during the transition to adolescence may provide the opportunity to buffer the adverse impact of COVID-19 on mental health. Translating such knowledge to inform intervention and prevention strategies for youths exposed to adversity is a critical goal for the field.

**Supplementary Information:**

The online version contains supplementary material available at 10.1186/s13034-023-00659-y.

## Introduction

Emerging studies have examined the relationship between pandemic-related disruptions and mental health in the general young population. Longitudinal studies and repeated cross-sectional studies have shown that depressive and anxiety symptoms, suicidal ideation, and suicide attempts increased from before to during the pandemic [2, 3, 29, 35, 40]. However, there is a scarcity of studies on vulnerable and underserved children, especially those born in socioeconomically vulnerable families in rural China and persistently separated from their parents, which have been largely unexplored.

An estimated 69 million children (25.4% of the child population) were experiencing parent–child separation in China, of which 40.51 million rural children are left behind by their parents’ migrating to urban areas to find work to provide for their families [30]. Social isolation and high physical and psychosocial deprivation increase their risk of mental health outcomes [11, 43]. In China, some migrant parents choose to return home when their children start school (around age 6) to support their children emotionally and help them succeed in school [16]. Previous studies have shown that children who experienced parent–child separation are at a high risk of developing psychological problems during the COVID-19 pandemic [34] and after lifting the restrictions [25]. In addition, the parent–child separation duration and timing may be a effective predictor of children’s mental problems. A recent study about 1177 left-behind adolescents’ loneliness revealed that the longer parents are separated from their children, the greater the negative impact on children [42]. An important issue that has been largely unexplored in the existing literature is the pattern (timing and duration) of separation and the effect on children’s mental and behavioral health domains. Given that the sensitivity of neural circuits to adversity undergoes dynamic changes from prenatal to young adulthood, the effects of parent–child separation may vary with the developmental stage at which separation occurs and how long it persists [12, 19]. Among pandemic-related research examining young people’s mental health issues such as depression, anxiety, and suicide related problems, we are yet to see longitudinal data to track the adverse effects on non-suicide self-injurious (NSSI), suicidal ideation, and suicide attempt. Given the increasing rates of NSSI and suicide over the past decade [10], especially among youth, it is concerning.

The current study therefore used a longitudinal sample from a feeder location for migrant employees in the countryside of southern China, with the overall aim of describing the short- and longer-term trajectories in mental health (depressive and anxious symptoms, NSSI, suicide-related problems) of school-aged children before and during the initial and later phases of the pandemic. We aimed to evaluate the differential performance of mental health in children with different experiences of parent–child separation to identify potential remedial windows; and attempted to identify additional risk and protective factors for mental health in response to the pandemic. Findings complement emerging literature [21, 31, 39] by providing insight into the changes in mental health before and during the pandemic among highly vulnerable and neglect-exposed children with parent–child separation experiences.

## Methods

### Sample

Data came from an ongoing representative longitudinal study of rural children living in Chizhou, Anhui Province. Children in grades 4 through 8 from local primary and junior high schools in Chizhou were recruited and followed up each year since October 2019. After 3 months of lockdown due to the COVID-19 pandemic, schools in Chizhou were reopened on April 26, 2020. An additional investigation was conducted 2 weeks after the school reopening in May 2020.

As illustrated in Additional file 1: Figure S1, of the original 1276 respondents who completed a pre-pandemic survey (Oct. 2019), 1236 participated in Wave 2 (May 2020), 1209 in Wave 3 (Oct. 2020), and 723 in Wave 4 (Oct. 2021).

### Measures

#### Outcomes

The measurements for each outcome were consistent across all four wave surveys. Depressive symptoms were calculated using the 33-item Mood and Feelings Questionnaire (MFQ) [7, 9]. A binary variable was created using a cutoff point of 27 [37]. Anxiety symptoms (generalized anxiety) were calculated by computing the mean item scores for this dimension of the Overanxious subscale of MacArthur HBQ [6, 40]. The baseline's 85th percentile (score 4.08 or higher) was used as the cut-off point for identifying the presence of anxiety symptoms. NSSI behaviors were measured using the 8-item non-suicidal self-injurious (NSSI) behavior questionnaire. A binary variable was defined as three or more NSSI behaviors being reported. Suicidal ideation, suicide plan, and suicide attempt were assessed with reference to the ‘middle school questionnaire’ of the 2013 Youth Risk Behaviour Surveillance System in the USA [8, 33]. Suicidal ideation was defined as a ‘yes’ in response to the question ‘Have you ever thought about killing yourself in the past 3 months?’. Suicide plan was defined as a ‘yes’ in response to the question, “During the past 3 months, did you make any plans to kill yourself?”. Suicide attempt was defined as a ‘yes’ in response to the question, ‘Have you ever tried to kill yourself in the past 3 months?’.

### Parent–child separation experiences

In the present study, parents or caregivers were asked if both of the children's parents had migrated to urban areas for work for more than 6 months per year during early childhood (from birth to 6 years old) and/or during the past years (after 6 years old). Four categories were generated based on the timing and duration of parent–child separation: (1) no separation; (2) early childhood separation before 6 years old; (3) recent separation after 6 years old; (4) prolonged separation since birth.

### Demographic characteristics

Household socioeconomic status (SES), positive childhood experiences (PCEs), neglect, lifestyle behaviors (sleep-related problems, physical activity, screen time before bedtime), and health-related indicators (self-perceived health) were derived from physical examinations or questionnaires (detailed in Additional file 1).

### Statistical analysis

Changes in mental health within individuals during the COVID-19 pandemic were examined using generalized estimating models. First, we tested the whole population-level changes in the probability of the binary depressive symptoms, NSSI, suicidal ideation, and suicide plan before and during the COVID-19 pandemic. We then regressed standardized outcomes scores on COVID-19 period indicators to enable direct comparisons across the six mental health outcomes. Second, we examined the interaction effects between the COVID-19 period and the parent–child separation experiences to understand whether and how mental health outcomes changes during the COVID-19 pandemic periods might vary across different parent–child separation groups. The effects parameters included group and time as categorical predictors, as well as the group × time interaction. The no-separation group was the reference level for group, and the baseline survey was the reference level for time. Finally, we tested the moderating effects of the sociodemographic and lifestyle characteristics by regressing each mental health outcome on each factor. Data management and analyses were conducted in Stata version 15.1.

## Results

Table 1 displays the characteristics of the analytical sample. Compared with those lost to follow-up due to graduation at Wave 4 (n = 486), participants included in the analytical model (n = 723) were slightly younger and had a lower percentage of parent–child separation experiences (see Additional file 1: Table S1). The mean (SD) age at Wave 4 (Oct. 2021) was 11.56 (1.35) years, and 514 (38.0%) were girls.

**Table 1:** Baseline characteristics of the participants included in the longitudinal study

Characteristic	Full sample		No separation (N = 426)	Childhood separation (N = 132)	Recent separation (N = 61)	Prolonged separation since birth (N = 104)	*P*
	Range	Mean (SD), number (%)	Mean (SD), number (%)	Mean (SD), number (%)	Mean (SD), number (%)	Mean (SD), number (%)	
Age	8.75–13.75	11.56 (1.17)	11.56 (1.20)	11.55 (1.28)	11.39 (1.20)	11.77 (0.87)	0.107
Female	0, 1	275 (38)	162 (38.0)	20 (32.8)	52 (39.4)	39.4 (41.0)	0.825
BMI	11.93–35.26	18.88 (3.49)	18.89 (3.41)	19.03 (3.80)	18.31 (3.19)	19.46 (3.93)	0.091
Positive childhood experiences	0–5	1.64 (1.12)	2.10 (1.41)	1.15 (1.26)	1.07 (1.85)	0.78 (1.43)	0.000
High neglect (upper quartile)	0, 1	18.4 (133)	14.8 (63)	16.4 (10)	19.7 (26)	32.7 (34)	0.000
Health status							0.530
Excellent		200 (27.9)	120 (28.1)	15 (24.6)	42 (31.8)	23 (22.1)	
Good		341 (46.2)	207 (48.6)	30 (49.5)	55 (41.7)	49 (47.1)	
Fair		165 (23.2)	88 (20.7)	16 (26.2)	31 (23.5)	30 (28.8)	
Poor		17 (2.7)	11 (2.6)	0 (0.0)	4 (3.0)	2 (1.9)	
Socioeconomic status							0.218
Low		57 (7.8)	59 (8.0)	1 (1.3)	12 (8.0)	10 (9.5)	
Moderate		488 (69.1)	291 (68.8)	48 (76.3)	80 (67.1)	69 (69.2)	
High		178 (23.1)	101 (23.2)	12 (22.5)	40 (24.9)	25 (21.3)	

At baseline (pre-pandemic level), 82 (11.3%) of 723 participants scored above the at-risk thresholds for depression, 85 (11.8%) for anxiety symptoms, 67 (9.3%) for NSSI, 137 (18.9%) for suicidal ideation, 53 (7.3%) for suicide plan, and 22 (3.0%) for suicide attempt (see Additional file 1: Table S2). Of the sample included, 132 (18.3%) were separated from their parents only during early childhood (before 6 years), 61 (8.4%) had recently separated from their parents after the age of 6, and 104 (14.4%) persistently separated from their parents since birth. In general, children who experienced parent–child separation tended to have poorer baseline mental health than those who had never been separated from their parents.

**Fig. 1:**
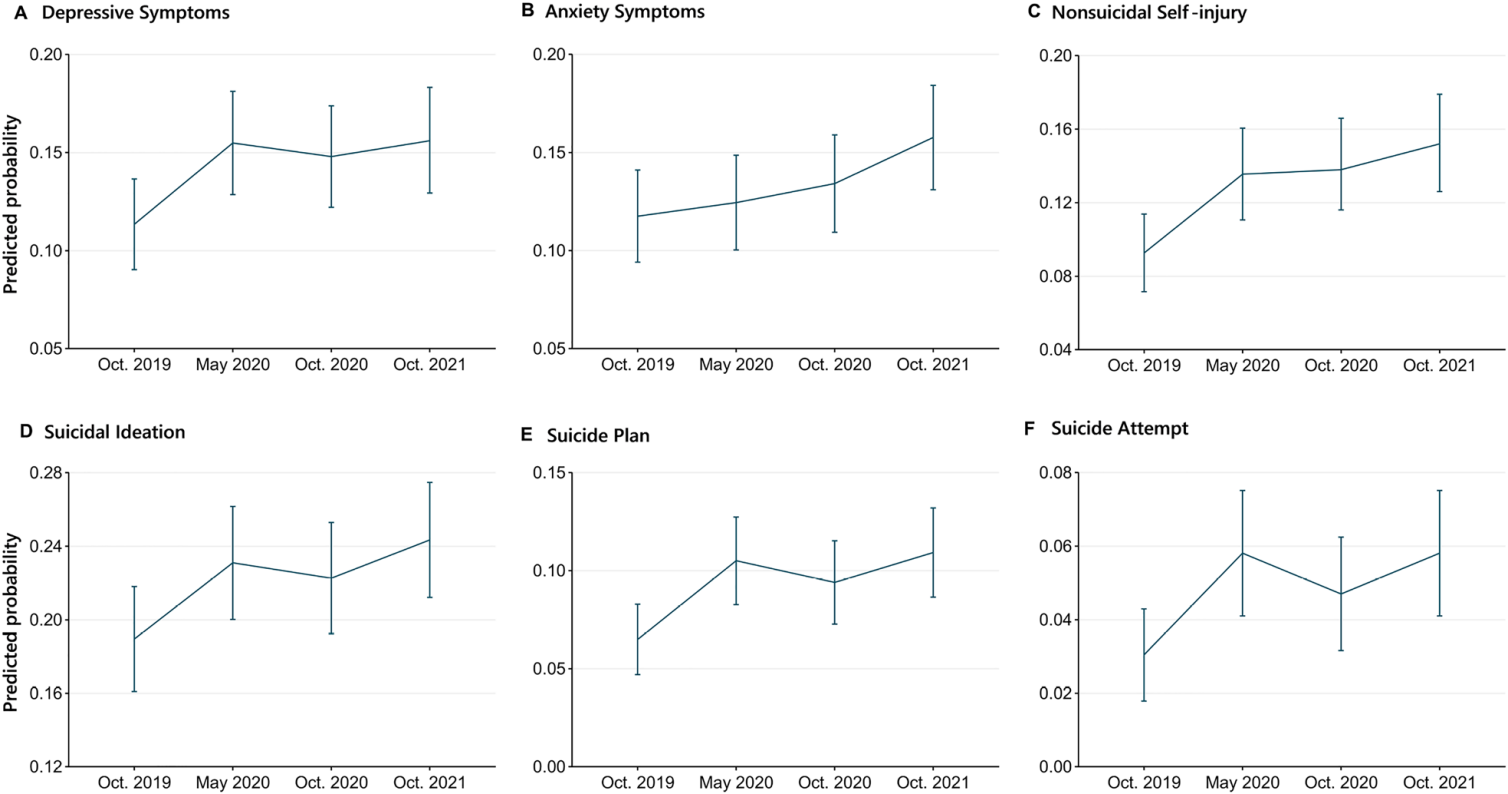
Predicted outcome trajectories before and during the COVID-19 pandemic of the total sample. Estimates are from generalized estimating models. Error bars indicate 95% CIs

Figure 1, Additional file 1: Tables S3 and S4 report the predicted trajectories of mental health outcomes before (October 2019) and during the initial and later phases of the COVID-19 pandemic for the entire population. Compared to pre-pandemic levels, all mental health outcomes except for anxiety symptoms deteriorated after 3-month school lockdown (change in May 2020 compared to pre-epidemic: 4.1% [3.8%, 4.5%] for depressive symptoms; 4.3% [3.9%, 4.7%] for NSSI; 4.1% [3.9%, 4.4%] for suicidal ideation; 4.0% [3.6%, 4.4%] for suicide plan; 2.8% [2.3%, 3.2%] for suicide attempt) and then slightly moderated after 5-month past the school-reopening (October 2020). But surprisingly, instead of further moderation, all mental health outcomes, including anxiety symptoms worsened again during the subsequent 12-month follow-up (Change in October 2021 compared to pre-epidemic: 4.3% (3.9%, 4.6%) for depressive symptoms; 4.0% (3.7%, 4.3%) for anxiety symptoms; 6.0% (5.4%, 6.5%) for non-suicidal self-injury; 5.4% (5.1%, 5.7%) for suicidal ideation; 4.4% (3.9%, 4.9%) for suicide plan; 2.8% (2.3%, 3.2%) for suicide attempt).

There may be a lagged long-term worsening effect of anxiety symptoms that gradually emerges over time; in the present study, anxiety symptoms did not show significant worsening at the time of school reopening and the subsequent 5-month follow-up, whereas when followed up to 19 months, the probability of anxiety symptoms exhibited a significant deterioration than before the pandemic (4.0% (3.7%, 4.3%]). Among the six mental health outcomes, NSSI showed the sharpest deterioration before and during the initial and later phases of the COVID-19 pandemic (Additional file 1: Figure S2, Table S5).

**Fig. 2:**
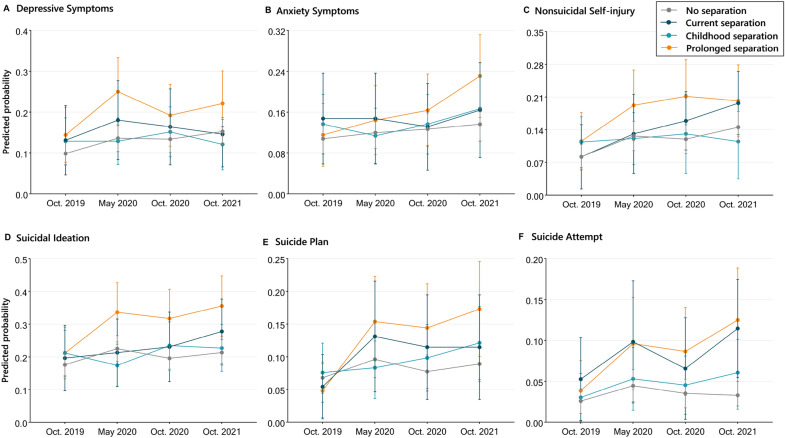
Interaction effects (time × group) between immediate and long-term changes in mental health and parent–child separation experiences

Figure 2 reports the predicted trajectories of mental health outcomes among children with parent–child separation experiences before (Wave 1) and during the initial and later phases of the COVID-19 pandemic. Compared to children never separated from their parents, children who persistently separated from both parents since birth experienced the sharpest immediate deterioration in all mental health outcomes (Table 2, depressive symptoms: *β* = 0.80, 95% *CI* [0.37, 1.24], *P* < 0.001; NSSI: *β* = 0.67, 95% *CI* [0.19, 1.15], *P* = 0.006; suicide ideation: *β* = 0.67, 95% *CI* [0.27, 1.07], *P* = 0.001; suicide plan: *β* = 0.77, 95% *CI* [0.26, 1.27], *P* = 0.003; suicide attempt: β = 1.04, 95% *CI* [0.35, 1.73], *P* = 0.003) after 3-month school lockdown (Wave 2); at the subsequent 19-month follow-up, children with persistent parent–child separation since birth continued to show more deterioration in all mental health outcomes (Wave 4, depressive symptoms: *β* = 0.63, 95% *CI* [0.15, 1.10], *P* = 0.01; anxiety symptoms: *β* = 0.76, 95% *CI* [0.30, 1.22], *P* = 0.001; NSSI: *β* = 0.69, 95% *CI* [0.20, 1.18], *P* = 0.002; suicide ideation: *β* = 0.79, 95% *CI* [0.37, 1.20], *P* < 0.001; suicide plan: *β* = 0.87, 95% *CI* [0.34, 1.40], *P* = 0.001; suicide attempt: β = 1.44, 95% *CI* [0.80, 2.09], *P* < 0.001).

**Table 2:** Interaction effects (time × group) between immediate and longer-term changes in mental health and parent–child separation experiences

Parent–child separation	Depressive symptoms				Anxiety symptoms				Non-suicidal self-injury				Suicidal ideation				Suicide plan				Suicide attempt			
	B	CI (lower)	CI (upper)	P	B	CI (lower)	CI (upper)	P	B	CI (lower)	CI (upper)	P	B	CI (lower)	CI (upper)	P	B	CI (lower)	CI (upper)	P	B	CI (lower)	CI (upper)	P
Before and May 2020																								
Change × no separation	Ref																							
Change × childhood	0.03	−0.45	0.51	0.902	−0.12	−0.64	0.39	0.635	0.13	−0.38	0.63	0.628	−0.21	−0.64	0.22	0.342	0.05	−0.52	0.63	0.138	0.45	−0.32	1.22	0.254
Change × recent	0.42	−0.19	1.04	0.176	0.14	−0.54	0.82	0.681	0.33	−0.35	1.01	0.336	0.07	−0.51	0.65	0.803	0.53	−0.17	1.22	0.855	1.01	0.13	1.89	**0.024**
Change × prolonged	0.80	0.37	1.24	**0.000**	0.23	−0.28	0.74	0.377	0.67	0.19	1.15	**0.006**	0.67	0.27	1.07	**0.001**	0.77	0.26	1.27	**0.003**	1.04	0.35	1.73	**0.003**
Before and Oct. 2020																								
Change × no separation	Ref																							
Change × childhood	0.23	−0.23	0.68	0.332	0.09	−0.41	0.58	0.733	0.35	−0.32	1.03	0.306	0.21	−0.19	0.61	0.298	0.29	−0.27	0.85	0.111	0.37	−0.46	1.19	0.386
Change × recent	0.31	−0.33	0.95	0.339	0.01	−0.73	0.74	0.987	0.47	0.01	0.92	**0.046**	0.21	−0.36	0.79	0.468	0.59	−0.14	1.32	0.311	0.63	−0.43	1.68	0.244
Change × prolonged	0.47	−0.01	0.94	0.054	0.36	−0.15	0.87	0.162	0.81	0.35	1.27	**0.001**	0.63	0.22	1.04	**0.002**	0.76	0.22	1.30	**0.006**	1.00	0.28	1.73	**0.007**
Before and Oct. 2021																								
Change × no separation	Ref																							
Change × childhood	−0.13	−0.91	0.64	0.733	0.31	−0.15	0.77	0.190	0.11	−0.65	0.86	0.784	−0.01	−0.64	0.62	0.972	0.48	−0.06	1.02	0.058	0.69	−0.06	1.44	0.072
Change × recent	0.19	−0.29	0.67	0.434	0.26	−0.41	0.94	0.445	0.66	0.22	1.11	**0.003**	0.40	−0.00	0.80	0.050	0.69	−0.02	1.41	0.080	1.30	0.45	2.14	**0.003**
Change × prolonged	0.63	0.15	1.10	**0.010**	0.76	0.30	1.22	**0.001**	0.69	0.20	1.18	**0.006**	0.79	0.37	1.20	**0.000**	0.87	0.34	1.40	**0.001**	1.44	0.80	2.09	**0.000**
Before vs during COVID-19 (average)																								
Change × no separation	Ref																							
Change × childhood	0.11	−0.23	0.45	0.512	0.09	−0.27	0.44	0.636	0.20	−0.30	0.70	0.432	0.12	−0.17	0.41	0.411	0.25	−0.15	0.65	**0.029**	0.46	−0.09	1.01	0.099
Change × recent	0.18	−0.30	0.65	0.464	0.12	−0.38	0.62	0.632	0.37	0.04	0.71	**0.029**	0.07	−0.35	0.49	0.744	0.57	0.06	1.08	0.218	0.95	0.31	1.60	**0.004**
Change × prolonged	0.59	0.26	0.93	**0.000**	0.45	0.10	0.81	**0.012**	0.66	0.31	1.01	**0.000**	0.67	0.38	0.96	**0.000**	0.77	0.38	1.15	**0.000**	1.12	0.63	1.62	**0.000**

^×^This indicates interaction effects between time changes in mental health and parent–child separation experience groups

Similarly, children who had recently separated from their parents after the age of 6 also exhibited greater longer-term deterioration in NSSI and suicide attempts (Wave 4, NSSI: *β* = 0.66, 95% *CI* [0.22, 1.11], *P* = 0.003; suicide attempt: β = 1.30, 95% *CI* [0.45, 2.24], *P* = 0.003). In contrast, children separated from both parents in early childhood (before age 6) and then reunited with parents after showed a similar or smaller magnitude of deterioration than children without parent–child separation.

Overall, the immediate and longer-term changes in all mental health outcomes for the full sample were largely driven by the deterioration in children who had been separated from their parents since birth; in addition, the longer-term changes in NSSI and suicide attempts were also explained by deterioration in children with the recent separation from both parents (after the age of 6 years).

**Fig. 3:**
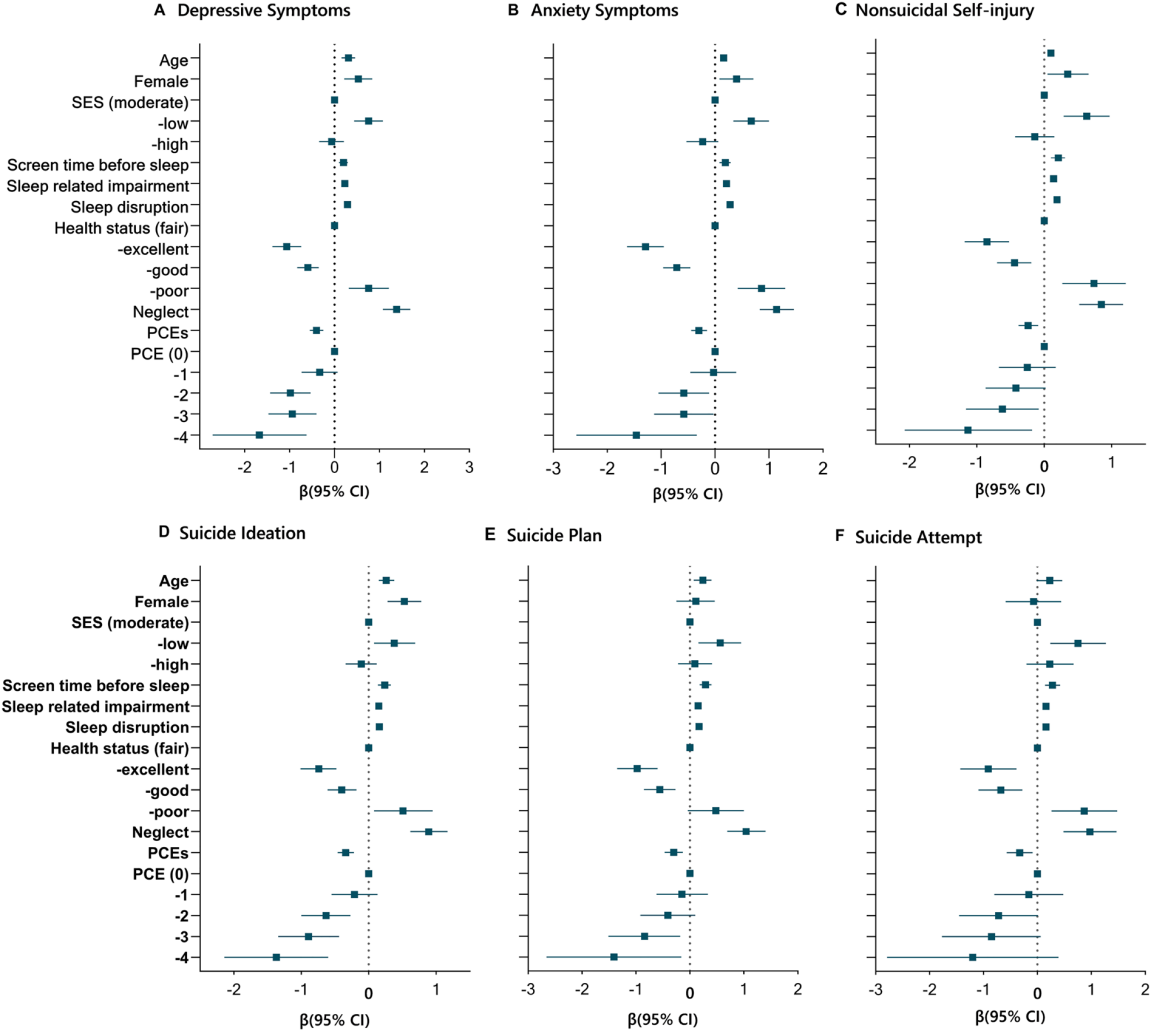
Moderating effect of the sociodemographic and lifestyle factors on continuous mental health outcomes. Coefficient plot of the point estimates and 95% CIs for the moderating role of sociodemographic and lifestyle characteristics. Estimates for socioeconomic and lifestyle characteristics are from generalized estimating models in which the binary mental health outcome (average changes from Wave 2 to 4) was regressed on this indicator with the variable listed in the vertical axis

Figure 3 reports the results from generalized estimating models for the moderating role of sociodemographic characteristics and lifestyle factors on the binary mental health outcomes before and during the pandemic. Being girls, greater age, low SES, more screen time before bed, sleep-related problems, and self-perceived poor health were associated with worse mental health outcomes (average changes from Wave 1 to 4). Notably, high economic status did not confer a significant protective effect on mental health during the epidemic, especially for suicide plan and attempt. Positive childhood experiences had a buffering effect on changes in mental health during the epidemic, and the effect values showed a dose–response trend (Additional file 1: Table S6).

## Discussion

This study responds to the urgent call for research to further understand the mental health effects of the COVID-19 pandemic across vulnerable groups [13]. This study provides population-based evidence, including multiple time points before and during the initial and later phases of the COVID-19 pandemic, to examine the short- and longer-term trajectories of mental health in the context of left-behind children in rural China. Our results indicated that adolescents’ mental health outcomes continue to worsen even 2 years after the COVID-19 outbreak, especially among those with prolonged parent–child separation experiences. This study also provides preliminary evidence that parent–child reunion during the transition to adolescence may provide buffering opportunities.

After 2 years of follow-up, approximately twice as many children separated from parents since birth experienced depressive symptoms, anxiety symptoms, and NSSI, and three times as many had suicide-related problems compared to the pre-pandemic levels, significantly higher than that in children without parent–child separation. Similarly, children who recently separated from their parents also exhibited greater deterioration (short- and longer-term) in NSSI and suicide attempts than children who had never been separated. However, children with childhood parent–child separation and reunited with parents during the transition to adolescence showed similar mental health trajectories to children who had never experienced parent–child separation. According to the attachment theory, the sense of attachment security might enable the children to better cope with stressors [5, 20]. When children are separated from their parents, they may experience risk for ongoing attachment disruptions [14] and develop an insecure attachment marked by avoidance or anxious-escalation of their attachment needs [20]. The insecure attachment is associated with poor mental health outcomes [18], especially during the COVID-19 pandemic [32]. It may explain worsening mental health outcomes among those who remained the children experiencing prolonged parent–child separation. It also highlights the importance of parental support for helping young children cope with stressors.

Our findings regarding differences in mental health changes across parent–child separation patterns during the COVID-19 pandemic are novel. These results suggest, first and foremost, that early adversity may have a recent and cumulative effect on mental health. The multiple challenges in such a fragile, insecure, and neglected setting increase the vulnerability of children experiencing recent and prolonged parent–child separation to mental health disruptions. Second, the resilient performance in response to the epidemic among children who were separated only until age six but reunited with their parents during the adolescent transition suggests that adolescence as a period of heightened neurobiological plasticity and sensitivity to the social environment [26, 28], may confer unique opportunities for buffering the harmful effects of early childhood through radical improvements in the rearing environment compared to earlier years, and thus potentially acquiring the ability to better cope with future stressors.

Several factors might play a part in the deteriorating mental health of these individuals over this period. For instance, our findings indicated that children with the perception of poor health, longer screen time before bed, and irregular sleep patterns were more likely to have increased psychological distress. Gender may be an additional source of disparities in the adverse psychological impact of the pandemic. Girls have experienced more negative changes in mental health than boys since the pandemic started. Sex-related differences in stress reactivity and resilience may partially account for more psychological distress in girls than boys [36]. The COVID-19 pandemic has further highlighted disparities in mental health burdens for girls [22, 24, 27, 36, 38]. Girls need to undertake more family work than boys, especially in rural [15]. The same was seen in chinese rural girls are left behind, more than one-third of them bear the responsibility for housework and childcare [41]. This labor burden imposes extra stress on girls apart from the COVID-related stressors. Notably, findings suggest that PCEs may mitigate the negative effect of the COVID-19 pandemic on mental health in a dose–response manner. These results are in line with the investigations among Wisconsin adults, indicating that PCEs largely buffered the effect of ACEs on adult mental health when both ACEs and PCEs in the same model [4].

One might hope the negative psychological impact of the COVID-19 pandemic on children and adolescents will be short-lived. However, this is not necessarily the case. Against the backdrop of more than 19 consecutive months of very low confirmed cases in China, psychological burden among children with recent and prolonged parent–child separation remained substantially high. During the last wave (Oct. 2021) of the present study, children reporting suicidal plans and attempts remained twice to three times higher than pre-pandemic levels. Notably, there may be a lagged and lasting effect of the COVID-19 pandemic on youth suicide, as summarized by Asarnow and Chung [1]. Time-limited adverse experiences may fundamentally change the way individuals take to challenges, especially in children and adolescents with developing brains. Furthermore, the interaction between lifestyle changes and psychosocial stress caused by health-related disasters could further aggravate the detrimental effects on children’s physical and mental health, which could cause a vicious cycle.

While mental health services have been provided to left-behind children by some local governments during the pandemic, most of the local governments were unable to afford more mental health services because of a lack of health resources, or even unaware of the problem. The present research provides essential evidence for policy makers and service planners. Based on the current study results, more attention should be paid to the mental health of rural children with parent–child separation experiences, especially during times of stress and crisis. Policy makers should consider this information and channel resources to target children in greatest need.

## Limitations

Our findings need to be interpreted in light of several limitations. These include using self-reported mental health outcomes rather than clinical diagnostic data. In several prior longitudinal samples, however, worsening depression and other mental health outcomes self-reported online or by telephone across different age groups correlated significantly with the COVID-19 pandemic [21, 23, 39], suggesting that repeated self-report measures adequately represented the changes of psychological status during the pandemic. Although we explored several potential determinants in this period, we lacked some factors that might give a more complete picture of mental health deterioration, such as parent–child conflict or exposure to violence and abuse. Despite our efforts to control the covariates that affect outcomes, multicollinearity has been recognized as principal difficulties in the present study. In addition, due to graduation from middle school, the response rate in the last survey was considerably lower than in the first three. Given that high school seniors may suffer the most during the pandemic [17], mental health deterioration in 2021 was largely underestimated.

## Conclusions

Our findings suggest that, among Chinese rural adolescents, parent–child separation experience was associated with deteriorated mental health outcomes during and after the COVID-19 outbreak. Furthermore, parent–child reunion during the transition to adolescence may provide buffering opportunities. Enhancing the quantity and quality of positive childhood experiences, including warm nurturing, and seizing the unique opportunity for caregiver buffering during adolescence may help steel children's resilience in the face of future stressors. Translating such knowledge to inform intervention and prevention strategies for youths exposed to adversity is a critical goal for the field.

### Supplementary Information

**Additional file 1: Figure S1.** Enrollment in the study and daily new confirmed COVID-19 cases. **Table S1.** Comparison of the baseline characteristics of participants included in the analytical sample vs those not included at wave 4. **Table S2.** Descriptive statistics by different parent–child separation experiences of the mental health outcomes before and during COVID-19. **Table S3.** Prevalence of mental health outcomes predicted from generalized estimation models before and during COVID-19. **Table S4.** Generalized estimating models: changes in mental health before and during the COVID-19 pandemic. **Figure S2.** Standardized Changes in Mental Health Before and During the COVID-19 Pandemic. **Table S5.** Generalized estimating models: standardized changes in mental health before and during the COVID-19 pandemic. **Table S6.** Generalized estimating models: interaction effects (time × group) between immediate and longer-term changes in mental health and parent–child separation experiences. **Table S7.** Moderating effect of socio-demography characteristics.

## Data Availability

The data-sets analyzed during this study are available from the corresponding author on reasonable request.

## Abbreviations

NSSI: Non-suicide self-injurious
ACE: Adverse childhood experiences
PCEs: Positive childhood experiences
