# Supplementary material for: Immediate and longer-term changes in mental health of children with parent–child separation experiences during the COVID-19 pandemic
Source: Child Adolesc Psychiatry Ment Health. 2023 Oct 4;17:113. doi: 10.1186/s13034-023-00659-y (PMC10552287; doi:10.1186/s13034-023-00659-y)
Supplement: Supplementary file 1 — Additional file 1: Figure S1. Enrollment in the study and daily new confirmed COVID-19 cases. Table S1. Comparison of the baseline characteristics of participants included in the analytical sample vs those not included at wave 4. Table S2. Descriptive statistics by different parent–child separation experiences of the mental health outcomes before and during COVID-19. Table S3. Prevalence of mental health outcomes predicted from generalized estimation models before and during COVID-19. Table S4. Generalized estimating models: changes in mental health before and during the COVID-19 pandemic. Figure S2. Standardized Changes in Mental Health Before and During the COVID-19 Pandemic. Table S5. Generalized estimating models: standardized changes in mental health before and during the COVID-19 pandemic. Table S6. Generalized estimating models: interaction effects (time × group) between immediate and longer-term changes in mental health and parent–child separation experiences. Table S7. Moderating effect of socio-demography characteristics. [file 13034_2023_659_MOESM1_ESM.docx]

**The Role of Adversity Timing in Mental Health of Children in Response to the COVID-19 Pandemic**

**Additional methods**

**Additional Fig 1.** Enrollment in the study and daily new confirmed COVID-19 cases.

**Additional Table 1.** Comparison of the baseline characteristics of participants included in the analytical sample vs those not included at wave 4.

**Additional Table 2.** Descriptive statistics by different parent-child separation experiences of the mental health outcomes before and during COVID-19.

**Additional Table 3.** Prevalence of mental health outcomes predicted from generalized estimation models before and during COVID-19.

**Additional Table 4.** Generalized estimating models: changes in mental health before and during the COVID-19 pandemic.

**Additional Fig 2.** Standardized Changes in Mental Health Before and During the COVID-19 Pandemic.

**Additional Table 5.** Generalized estimating models: standardized changes in mental health before and during the COVID-19 pandemic.

**Additional Table 6.** Generalized estimating models: interaction effects (time×group) between immediate and longer-term changes in mental health and parent-child separation experiences.

**Additional Table 7.** Moderating effect of socio-demography characteristics.

**Additional methods**

**Sociodemographic Characteristics**

**Socioeconomic status (SES)**

Children were asked to describe subjective perceptions of the family’s SES with five response alternatives: “very low”, “low”, “moderate”, “high”, and “very high”. The five responses were clustered into three categories: low (“very low” and “low”), moderate, and high (“high” and “very high”).

**Sleep-related problems**

Sleep disturbance and sleep-related impairment experienced in the past month were measured using Patient-reported Outcomes Measurement Information System (PROMIS) 8-item short forms (Forrest et al., 2018; Bevans et al., 2019; Fishbein et al., 2020). All items were scored on a 5-point scale (1 = not at all or never; 2 = a little bit or rarely; 3 = somewhat or sometimes; 4 = quite slightly or often; 5 = very much or always), in which higher scores indicate more serious problems.

Sleep disturbance items capture sleep continuity, sleep onset, parasomnias, and sleep quality, while sleep-related impairment captures the energy, daytime sleepiness, sleep offset, and the impact of sleep on behavior, cognitive function, affect, and daily activities. The scale demonstrated good internal consistency in the current sample (α= 0.87).

**Physical activity**

Physical activity levels were ascertained from the Youth Risk Behavior Survey 2015 (Kann et al., 2016) at each wave. Adolescents were asked, “During the past 7 days, on how many days were you physically active for more than 60 minutes per day?”.

**Screen time before sleep**

Average screen time before sleep per day was assessed through the question ‘In the past 7 days, how many times did you spend on your computer, tablet (iPad), mobile phone, video game console before sleep?’ And are scored on a 5-point scale consisting of 0 (“no”), 1 (“<1 hour/day”), 2 (“1-2 hours/day”), 3 (“2-3 hours/day”), 4 (“3-4 hours/day”), and 5 (“>5 hours/day”).

**Self-reported health**

Perceived health status was assessed although the question ‘In general, how do you feel about your health?’ In addition, vital capacity, heart rate, systolic blood pressure, and caries were measured by professional medical workers using appropriate instruments during the health checkup.

**Neglect**

Adolescents reported neglect experience through the 10-item Adverse Childhood Experiences Questionnaire-Short Form (ACES-SF) (Felitti et al., 1998; Meinck et al., 2017). The number of affirmative responses of the following four questions ‘Did you often or very often feel that: 1) no one in your family loved you or thought you were important or special; 2) your family didn’t look out for each other, feel close to each other, or support each other; 3) you didn’t have enough to eat, had to wear dirty clothes, and had no one to protect you; 4) your parents were too drunk or high to take care of you or take you to the doctor if you needed it?’ were summed, and a binary variable was created using the 75th percentile (score 2 or higher) as the cut-off point for identifying the higher presence of neglect experiences.

**Positive childhood experiences (PCEs)**

PCEs variables were measured in wave 3. According to previous literature, we created a PCEs index based on the presence or absence of five components: high parental education, high perceived SES, high parental warmth, two-parent family, and high peer support. Then, the total number of PCEs categories that a child was exposed to are summed to create a PCEs score ranging from 0 to 5.

***High parental education.*** Parental education was categorized into three groups: (1) "< middle school", (2) "high school", and (3) "≥ college". Participants who reported that the highest level of parental education (using the highest value for either mother or father) was a college degree or higher were considered "high parental education" (Slopen et al., 2017).

***High perceived SES.***  Participants reported on their socioeconomic status during childhood compared to the average family. Responses ranged from 1 (very poor) to 5 (very good). Participants who reported good or very good were considered as “high perceived SES” (Slopen et al., 2017).

***Two-parent family.*** Family structure was queried with a simple question: “Which of the following is your family type?” Participants who responded live with both of biological parents were categorized as growing up in two-parent family (Slopen et al., 2017).

***High parental warmth.***  Parental warmth was queried with a 13-item scale adapted from Self-Reported Parenting Attitudes and Behaviors Scale scored on a five-point Likert scale ranging from strongly disagree to strongly agree (Raudino et al., 2012). Responses were coded such that a higher score represents greater warmth (α = 0.84). We created quartiles of the score, and participants in the top quartile were categorized as having high parental warmth (Slopen et al., 2017).

***High peer support.*** We created an indicator for high peer support (using the number of good friends), whereby three or more were considered as high (Sheikh, 2018).

**References**

1. Meinck, F., Cosma, A. P., Mikton, C., Baban, A., 2017 . Psychometric properties of the Adverse Childhood Experiences Abuse Short Form (ACE-ASF) among Romanian high school students. Child Abuse Negl. 72, 326–337. https://doi.org/10.1016/j.chiabu.2017.08.016
2. Raudino A, Woodward LJ, Fergusson DM, Horwood LJ., 2012. Childhood conduct problems are associated with increased partnership and parenting difficulties in adulthood. *J Abnorm Child Psychol*. 40(2):251-263.
3. Sheikh M. A., 2018. The potential protective effect of friendship on the association between childhood adversity and psychological distress in adulthood: A retrospective, preliminary, three-wave population-based study. J Affect Disord. 226, 21–27. https://doi.org/10.1016/j.jad.2017.09.015
4. Slopen, N., Chen, Y., Guida, J. L., Albert, M. A., & Williams, D. R., 2017. Positive childhood experiences and ideal cardiovascular health in midlife: Associations and mediators. Preventive medicine. 97, 72–79. https://doi.org/10.1016/j.ypmed.2017.01.002
5. Felitti, V. J., Anda, R. F., Nordenberg, D., Williamson, D. F., Spitz, A. M., Edwards, V., Koss, M. P., Marks, J. S., 1998. Relationship of childhood abuse and household dysfunction to many of the leading causes of death in adults. The Adverse Childhood Experiences (ACE) Study. Am J Prev Med. 14(4), 245–258. <https://doi.org/10.1016/s0749-3797(98)00017-8>
6. Meinck, F., Cosma, A. P., Mikton, C., & Baban, A., 2017. Psychometric properties of the Adverse Childhood Experiences Abuse Short Form (ACE-ASF) among Romanian high school students. Child Abuse Negl. 72, 326–337. https://doi.org/10.1016/j.chiabu.2017.08.016

**
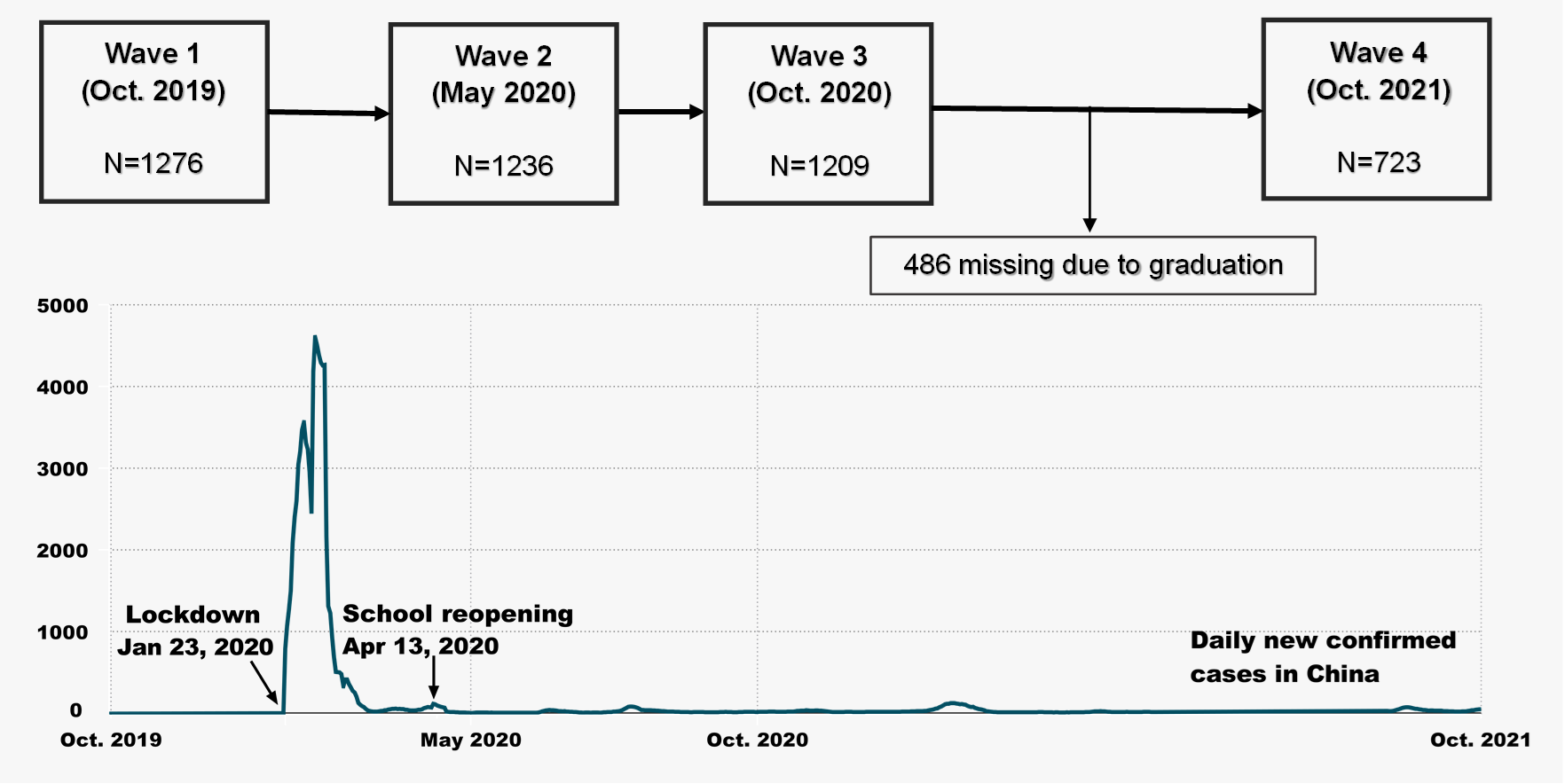
 Additional Fig 1.** Enrollment in the study and daily new confirmed COVID-19 cases.

Embedded figure: Daily new confirmed COVID-19 cases. 7-day rolling average. Due to limited testing, the number of confirmed cases is lower than the true number of I infections. Source: Johns Hopkins University CSSE COVID-19 Data.

**Additional Table 1.** Comparison of the baseline characteristics of participants included in the analytical sample vs those not included at wave 4.

|  | **Non-included (N=486)** | **Included (N=723)** | **p-value** |
| --- | --- | --- | --- |
| **Age** |  |  | **.001** |
| Mean (SD) | 12.61 (1.36) | 11.56 (1.16) |  |
| Range | 9.0-14.0 | 8.67-17.34 |  |
| **Sex** |  |  | .061 |
| Male | 314 (56.8%) | 448 (62.0%) |  |
| Female | 239 (43.2%) | 275 (38.0%) |  |
| **BMI** |  |  | .184 |
| Mean (SD) | 19.33 (3.22) | 18.84 (3.47) |  |
| Range | 13.57-32.03 | 11.93-35.26 |  |
| **Health status** |  |  | .346 |
| Excellent | 134 (25.7%) | 200 (29.7%) |  |
| Good | 244 (46.8%) | 308 (45.7%) |  |
| Fair | 126 (24.2%) | 151 (22.4%) |  |
| Poor | 17 (3.3%) | 15 (2.2%) |  |
| **Parent-child separation** |  |  | **.029** |
| No separation | 350 (65.3%) | 426 (59.0%) |  |
| Separation after 6 yrs | 25 (4.7%) | 61 (8.4%) |  |
| Separation during 0-6 yrs | 90 (16.8%) | 131 (18.1%) |  |
| Separation from birth to date | 71 (13.2%) | 104 (14.4%) |  |
| **Socioeconomic status^δ^** |  |  | 0.551 |
| Low | 43 (8.2%) | 51(7.5%) |  |
| Moderate | 369 (70.2%) | 464(68.2%) |  |
| High | 114(21.7) | 165(24.3) |  |
| **Screen time before sleep, hours** |  |  | **<0.001** |
| Mean (SD) | 0.61 (1.39) | 0.30 (0.83) |  |
| Range | 0-6 | 0-6 |  |
| **Midnight snack, days/week** |  |  | 0.606 |
| Mean (SD) | 2.57 (1.91) | 2.51 (1.93) |  |
| Range | 0-5 | 0-5 |  |

Data are mean (SD), number (%).

**Additional Table 2.** Descriptive statistics by different parent-child separation experiences of the mental health outcomes before and during COVID-19.

|  | **Total** | **No separation**  **(N=426)** | **Early childhood separation (N=132)** | **Recent separation (N=61)** | **Prolonged separation since birth (N=104)** |
| --- | --- | --- | --- | --- | --- |
| **Depressive symptoms (yes/no)** | **%(n)** | **%(n)** | **%(n)** | **%(n)** | **%(n)** |
| Wave 1 (Oct. 2019) | 11.3 (82) | 9.9 (42) | 12.9 (17) | 13.1 (8) | 14.4 (15) |
| Wave 2 (May 2020) | 15.2 (112) | 13.6 (58) | 12.9 (17) | 18.0 (11) | 25.0 (26) |
| Wave 3 (Oct. 2020) | 14.8 (107) | 13.4 (57) | 15.2 (20) | 16.4 (10) | 19.2 (20) |
| Wave 4 (Oct. 2021) | 15.6 (113) | 15.3 (65) | 12.1 (16) | 14.6 (9) | 22.1 (23) |
| **Anxiety symptoms (yes/no)** |  |  |  |  |  |
| Wave 1 (Oct. 2019) | 11.8 (85) | 10.8 (46) | 13.6 (18) | 14.8 (9) | 11.5 (12) |
| Wave 2 (May 2020) | 12.4 (90) | 12.0 (51) | 11.4 (15) | 14.8 (9) | 14.4 (15) |
| Wave 3 (Oct. 2020) | 13.4 (97) | 12.7 (54) | 13.6 (18) | 13.1 (8) | 16.3 (17) |
| Wave 4 (Oct. 2021) | 15.8 (114) | 13.6(58) | 16.7 (22) | 16.4 (10) | 23.3 (24) |
| **Non-suicidal self-injury (yes/no)** |  |  |  |  |  |
| Wave 1 (Oct. 2019) | 9.3 (67) | 8.2 (35) | 11.4 (15) | 8.2 (5) | 11.5 (12) |
| Wave 2 (May 2020) | 13.6 (98) | 12.7 (54) | 12.1 (16) | 13.1 (8) | 19.2 (20) |
| Wave 3 (Oct. 2020) | 13.8 (100) | 12.0 (51) | 13.1 (17) | 16.4 (10) | 21.2 (22) |
| Wave 4 (Oct. 2021) | 15.2 (110) | 14.6 (62) | 11.5 (15) | 19.7 (12) | 20.2 (21) |
| **Suicidal ideation (yes/no)** |  |  |  |  |  |
| Wave 1 (Oct. 2019) | 18.9 (137) | 17.6 (75) | 21.2 (28) | 19.7 (12) | 21.2 (22) |
| Wave 2 (May 2020) | 24.1 (174) | 22.5 (96) | 17.4 (23) | 21.3 (13) | 33.7.4 (35) |
| Wave 3 (Oct. 2020) | 22.2 (161) | 19.7 (83) | 23.5 (31) | 23.3 (14) | 31.7 (33) |
| Wave 4 (Oct. 2021) | 24.2 (175) | 21.4 (91) | 22.7 (30) | 27.8 (17) | 35.6 (37) |
| **Suicide plan (yes/no)** |  |  |  |  |  |
| Wave 1 (Oct. 2019) | 7.3 (53) | 6.8 (29) | 7.6 (10) | 8.2 (5) | 8.7 (9) |
| Wave 2 (May 2020) | 10.7 (77) | 9.6 (41) | 8.3 (11) | 13.1 (8) | 16.3 (17) |
| Wave 3 (Oct. 2020) | 9.7 (70) | 7.7 (33) | 9.8 (13) | 13.1 (8) | 15.4 (16) |
| Wave 4 (Oct. 2021) | 11.2 (81) | 8.9 (38) | 12.1 (16) | 14.8 (9) | 17.3 (18) |
| **Suicide attempt (yes/no)** |  |  |  |  |  |
| Wave 1 (Oct. 2019) | 3.0 (22) | 2.6 (11) | 3.0 (4) | 4.9 (3) | 3.8 (4) |
| Wave 2 (May 2020) | 5.8 (42) | 4.5 (19) | 5.3 (7) | 9.8 (6) | 9.6 (10) |
| Wave 3 (Oct. 2020) | 4.7 (34) | 3.5 (15) | 4.5 (6) | 6.6 (4) | 8.7 (9) |
| Wave 4 (Oct. 2021) | 5.8 (42) | 3.3 (14) | 6.1 (8) | 11.5 (7) | 12.5 (13) |

**Additional Table 3.** Prevalence of mental health outcomes predicted from generalized estimation models before and during COVID-19.

|  | **Total** | **No separation**  **(N=426)** | **Early childhood separation (N=132)** | **Recent separation (N=61)** | **Prolonged separation since birth (N=104)** |
| --- | --- | --- | --- | --- | --- |
| **Depressive symptoms** | **%(95%CI)** | **%(95%CI)** | **%(95%CI)** | **%(95%CI)** | **%(95%CI)** |
| Wave 1 (Oct. 2019) | 11.3% (9.0%, 13.7%) | 9.9% (7.0%, 12.7%) | 12.9% (7.2%, 18.6%) | 13.1% (4.6%, 21.6%) | 14.4% (7.7%, 21.2%) |
| Wave 2 (May 2020) | 15.5% (12.9%, 18.1%) | 13.6% (10.4%, 16.9%) | 12.9% (7.2%, 18.6%) | 18.0% (8.4%, 27.7%) | 25.0% (16.7%, 33.3%) |
| Wave 3 (Oct. 2020) | 14.8% (12.2%, 17.4%) | 13.4% (10.1%, 16.6%) | 15.2% (9.0%, 21.3%) | 16.4% (7.1%, 25.7%) | 19.2% (11.7%, 26.8%) |
| Wave 4 (Oct. 2021) | 15.6% (12.9%, 18.3%) | 15.3% (11.8%, 18.7%) | 12.1% (5.9%, 18.2%) | 14.6% (6.6%, 16.4%) | 22.1% (14.1%, 30.1%) |
| **Anxiety symptoms** |  |  |  |  |  |
| Wave 1 (Oct. 2019) | 11.8% (9.4%, 14.1%) | 10.8% (7.9%, 13.7%) | 13.6% (7.8%, 19.5%) | 14.8% (5.9%, 23.7%) | 11.5% (5.4%, 17.7%) |
| Wave 2 (May 2020) | 12.4% (10.0%, 14.9%) | 12.0% (8.9%, 15.1%) | 11.4% (5.9%, 16.8%) | 14.8% (5.9%, 23.7%) | 14.4% (7.7%, 21.2%) |
| Wave 3 (Oct. 2020) | 13.4% (10.9%, 15.9%) | 12.7% (9.5%, 15.8%) | 13.6% (7.8%, 19.5%) | 13.1% (4.6%, 21.6%) | 16.3% (9.2%, 23.5%) |
| Wave 4 (Oct. 2021) | 15.8% (13.1%, 18.4%) | 13.6% (10.4%, 16.9%) | 16.7% (10.3%, 23.0%) | 16.4% (7.1%, 25.7%) | 23.1% (15.0%, 31.2%) |
| **Non-suicidal self-injury** |  |  |  |  |  |
| Wave 1 (Oct. 2019) | 9.3% (7.2%, 11.4%) | 8.2% (5.6%, 10.8%) | 11.4% (5.9%, 16.8%) | 8.2% (1.3%, 15.1%) | 11.5% (5.4%, 17.7%) |
| Wave 2 (May 2020) | 13.6% (11.1%, 16.0%) | 12.7% (9.5%, 15.8%) | 12.1% (6.6%, 17.7%) | 13.1% (4.6%, 21.6%) | 19.2% (11.7%, 26.8%) |
| Wave 3 (Oct. 2020) | 13.8% (11.6%, 16.6%) | 12.0% (8.9%, 15.1%) | 13.1% (4.6%, 21.6%) | 15.9% (9.7%, 22.1%) | 21.2% (13.3%, 29.0%) |
| Wave 4 (Oct. 2021) | 15.2% (12.6%, 17.9%) | 14.6% (11.2%, 17.9%) | 11.5% (3.5%, 19.5%) | 19.7% (12.9%, 26.5%) | 20.2% (12.5%, 27.9%) |
| **Suicidal ideation** |  |  |  |  |  |
| Wave 1 (Oct. 2019) | 18.9% (16.1%, 21.8%) | 17.6% (14.0%, 21.2%) | 21.2% (14.2%, 28.2%) | 19.7% (9.7%, 29.6%) | 21.2% (13.3%, 29.0%) |
| Wave 2 (May 2020) | 23.1% (20.0%, 26.2%) | 22.5% (18.6%, 26.5%) | 17.4% (11.0%, 23.9%) | 21.3% (11.0%, 31.6%) | 33.7% (24.6%, 42.7%) |
| Wave 3 (Oct. 2020) | 22.3% (19.2%, 25.3%) | 19.6% (15.8%, 23.4%) | 23.5% (16.3%, 30.7%) | 23.1% (12.4%, 33.7%) | 31.7% (22.8%, 40.7%) |
| Wave 4 (Oct. 2021) | 24.3% (21.2%, 27.5%) | 21.4% (17.5%, 25.3%) | 22.7% (15.6%, 30.3%) | 27.8% (17.8%, 37.7%) | 35.6% (26.4%, 44.8%) |
| **Suicide plan** |  |  |  |  |  |
| Wave 1 (Oct. 2019) | 6.5% (4.7%, 8.3%) | 6.8% (4.4%, 9.2%) | 7.6% (3.1%, 12.1%) | 5.4% (0.5%, 10.3%) | 4.8% (0.7%, 8.9%) |
| Wave 2 (May 2020) | 10.5% (8.3%, 12.7%) | 9.6% (6.8%, 12.4%) | 8.3% (3.6%, 13.0%) | 13.1% (4.6%, 21.6%) | 15.4% (8.5%, 22.3%) |
| Wave 3 (Oct. 2020) | 9.4% (7.3%, 11.5%) | 7.7% (5.2%, 10.3%) | 9.8% (4.8%, 14.9%) | 11.5% (3.5%, 19.5%) | 14.4% (7.7%, 21.2%) |
| Wave 4 (Oct. 2021) | 10.9% (8.7%, 13.2%) | 8.9% (6.2%, 11.6%) | 12.1% (6.6%, 17.7%) | 11.5% (3.5%, 19.5%) | 17.3% (10.0%, 24.6%) |
| **Suicide attempt** |  |  |  |  |  |
| Wave 1 (Oct. 2019) | 3.0% (1.8%, 4.3%) | 2.6% (1.1%, 4.1%) | 3.0% (0.1%, 6.0%) | 5.3% (0.2%, 10.3%) | 3.8% (0.2%, 7.5%) |
| Wave 2 (May 2020) | 5.8% (4.1%, 7.5%) | 4.5% (2.5%, 6.4%) | 5.3% (1.5%, 9.1%) | 9.8% (2.4%, 17.3%) | 9.6% (3.9%, 15.3%) |
| Wave 3 (Oct. 2020) | 4.7% (3.2%, 6.2%) | 3.5% (1.8%, 5.3%) | 4.5% (1.0%, 8.1%) | 6.6% (0.3%, 12.8%) | 8.7% (3.3%, 14.1%) |
| Wave 4 (Oct. 2021) | 5.8% (4.1%, 7.5%) | 3.3% (1.6%, 5.0%) | 6.1% (2.0%, 10.1%) | 11.5% (5.5%, 17.5%) | 12.5% (6.1%, 18.9%) |

**Additional Table 4.** Generalized estimating models: changes in mental health before and during the COVID-19 pandemic.

|  | β | SE | P | CI (lower) | CI (upper) |
| --- | --- | --- | --- | --- | --- |
| **Before and May 2020** |  |  |  |  |  |
| Depressive symptoms | 0.36 | 0.12 | **0.003** | 0.12 | 0.60 |
| Anxiety symptoms | 0.07 | 0.13 | 0.612 | -0.19 | 0.32 |
| Non-suicidal self-injury | 0.43 | 0.14 | **0.002** | 0.16 | 0.70 |
| Suicidal ideation | 0.25 | 0.11 | **0.018** | 0.04 | 0.46 |
| Suicide plan | 0.52 | 0.16 | **0.001** | 0.21 | 0.84 |
| Suicide attempt | 0.68 | 0.22 | **0.002** | 0.24 | 1.11 |
| **Before and Oct. 2020** |  |  |  |  |  |
| Depressive symptoms | 0.31 | 0.12 | **0.012** | 0.07 | 0.54 |
| Anxiety symptoms | 0.15 | 0.13 | 0.231 | -0.10 | 0.40 |
| Non-suicidal self-injury | 0.48 | 0.14 | **0.000** | 0.21 | 0.74 |
| Suicidal ideation | 0.20 | 0.11 | 0.056 | -0.01 | 0.41 |
| Suicide plan | 0.40 | 0.17 | **0.015** | 0.08 | 0.72 |
| Suicide attempt | 0.45 | 0.23 | **0.047** | 0.01 | 0.90 |
| **Before and Oct. 2021** |  |  |  |  |  |
| Depressive symptoms | 0.39 | 0.12 | **0.001** | 0.15 | 0.63 |
| Anxiety symptoms | 0.34 | 0.12 | **0.006** | 0.10 | 0.58 |
| Non-suicidal self-injury | 0.63 | 0.13 | **0.000** | 0.36 | 0.89 |
| Suicidal ideation | 0.32 | 0.10 | **0.002** | 0.11 | 0.52 |
| Suicide plan | 0.57 | 0.16 | **0.000** | 0.25 | 0.88 |
| Suicide attempt | 0.68 | 0.22 | **0.002** | 0.24 | 1.11 |


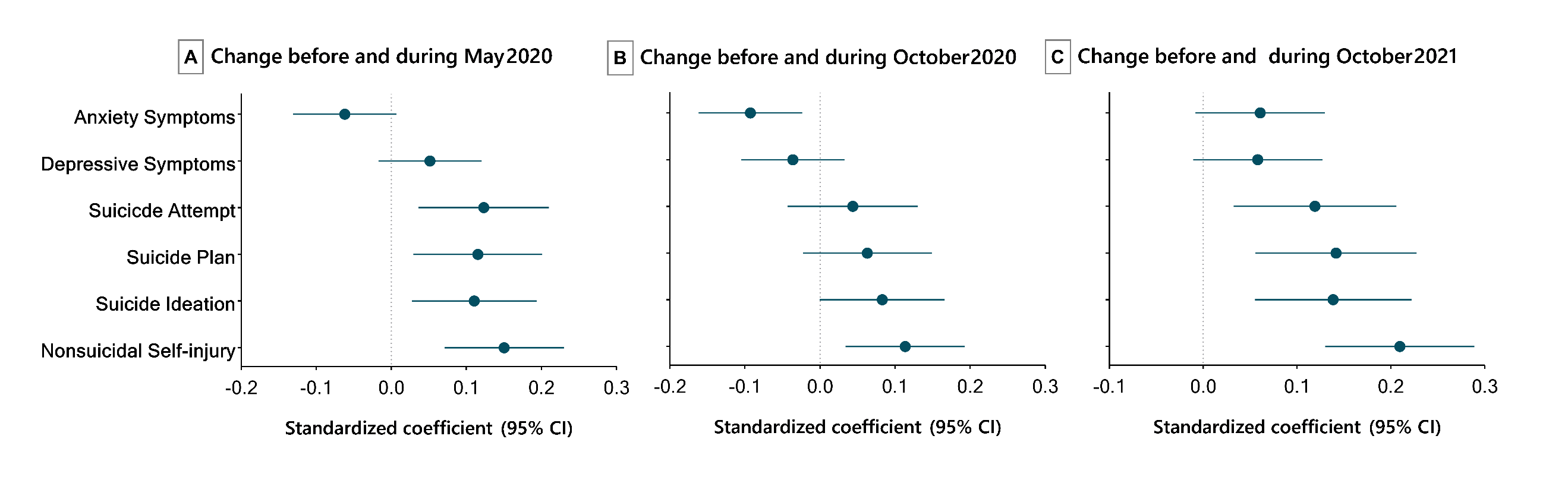


**Fig. 1** Standardized Changes in Mental Health Before and During the COVID-19 Pandemic.

Estimates are from Generalized estimating models (GEE). Error bars indicate 95% CIs. Continuous scores of all mental health outcomes were standardized and included in the analysis.

**Additional Table 5.** Generalized estimating models: standardized changes in mental health before and during the COVID-19 pandemic.

|  | β | SE | P | CI (lower) | CI (upper) |
| --- | --- | --- | --- | --- | --- |
| **Before and May 2020** |  |  |  |  |  |
| Depressive symptoms | 0.05 | 0.04 | 0.141 | -0.02 | 0.12 |
| Anxiety symptoms | -0.06 | 0.04 | 0.079 | -0.13 | 0.01 |
| Non-suicidal self-injury | 0.15 | 0.04 | **0.000** | 0.07 | 0.23 |
| Suicidal ideation | 0.11 | 0.04 | **0.009** | 0.03 | 0.19 |
| Suicide plan | 0.12 | 0.04 | **0.008** | 0.03 | 0.20 |
| Suicide attempt | 0.14 | 0.04 | **0.001** | 0.06 | 0.23 |
| **Before and Oct. 2020** |  |  |  |  |  |
| Depressive symptoms | -0.04 | 0.04 | 0.302 | -0.11 | 0.03 |
| Anxiety symptoms | -0.09 | 0.04 | **0.008** | -0.16 | -0.02 |
| Non-suicidal self-injury | 0.11 | 0.04 | **0.005** | 0.03 | 0.19 |
| Suicidal ideation | 0.08 | 0.04 | 0.051 | -0.00 | 0.17 |
| Suicide plan | 0.06 | 0.04 | 0.151 | -0.02 | 0.15 |
| Suicide attempt | 0.07 | 0.04 | 0.128 | -0.02 | 0.15 |
| **Before and Oct. 2021** |  |  |  |  |  |
| Depressive symptoms | 0.06 | 0.04 | 0.097 | -0.01 | 0.13 |
| Anxiety symptoms | 0.06 | 0.04 | 0.084 | -0.01 | 0.13 |
| Non-suicidal self-injury | 0.21 | 0.04 | **0.000** | 0.13 | 0.29 |
| Suicidal ideation | 0.14 | 0.04 | **0.001** | 0.06 | 0.22 |
| Suicide plan | 0.14 | 0.04 | **0.001** | 0.06 | 0.23 |
| Suicide attempt | 0.12 | 0.04 | **0.006** | 0.04 | 0.21 |

Standardized outcome scores were used in generalized estimating models to enable direct comparisons across the outcomes.

**Additional Table 6.** Moderating effect of socio-demography characteristics.

|  | **Depressive symptoms** | | | | **Anxiety symptoms** | | | | **Non-suicidal self-injury** | | | | **Suicidal ideation** | | | | **Suicide plan** | | | | **Suicide attempt** | | | |
| --- | --- | --- | --- | --- | --- | --- | --- | --- | --- | --- | --- | --- | --- | --- | --- | --- | --- | --- | --- | --- | --- | --- | --- | --- |
| **Characteristics** | β | CI  (lower) | CI  (upper) | P | β | CI  (lower) | CI  (upper) | P | β | CI  (lower) | CI  (upper) | P | β | CI  (lower) | CI  (upper) | P | β | CI  (lower) | CI  (upper) | P | β | CI  (lower) | CI  (upper) | P |
| **Age** | 0.31 | 0.16 | 0.46 | **0.000** | 0.16 | 0.10 | 0.21 | **0.000** | 0.10 | 0.08 | 0.12 | **0.000** | 0.26 | 0.15 | 0.38 | **0.000** | 0.24 | 0.07 | 0.40 | **0.005** | 0.23 | -0.01 | 0.46 | 0.062 |
| **Female** | 0.53 | 0.22 | 0.84 | **0.001** | 0.40 | 0.08 | 0.71 | **0.013** | 0.35 | 0.05 | 0.66 | **0.023** | 0.53 | 0.28 | 0.78 | **0.000** | 0.11 | -0.25 | 0.46 | 0.555 | -0.07 | -0.59 | 0.44 | 0.778 |
| **SES (moderate)** | Ref. |  |  |  |  |  |  |  |  |  |  |  |  |  |  |  |  |  |  |  |  |  |  |  |
| Low | 0.76 | 0.44 | 1.08 | **0.000** | 0.67 | 0.34 | 1.00 | **0.000** | 0.63 | 0.29 | 0.97 | **0.000** | 0.38 | 0.08 | 0.69 | **0.014** | 0.56 | 0.16 | 0.95 | **0.006** | 0.75 | 0.24 | 1.27 | **0.004** |
| High | -0.06 | -0.34 | 0.21 | 0.649 | -0.23 | -0.53 | 0.06 | 0.121 | -0.14 | -0.43 | 0.15 | 0.347 | -0.11 | -0.34 | 0.12 | 0.366 | 0.09 | -0.22 | 0.41 | 0.561 | 0.23 | -0.20 | 0.67 | 0.291 |
| **Screen time** | 0.20 | 0.10 | 0.30 | **0.000** | 0.19 | 0.08 | 0.29 | **0.001** | 0.21 | 0.10 | 0.31 | **0.000** | 0.24 | 0.14 | 0.33 | **0.000** | 0.29 | 0.18 | 0.40 | **0.000** | 0.28 | 0.14 | 0.42 | **0.000** |
| **S impairment** | 0.23 | 0.20 | 0.26 | **0.000** | 0.21 | 0.18 | 0.25 | **0.000** | 0.14 | 0.11 | 0.17 | **0.000** | 0.15 | 0.12 | 0.18 | **0.000** | 0.15 | 0.12 | 0.18 | **0.000** | 0.16 | 0.11 | 0.20 | **0.000** |
| **Sleep disruption** | 0.29 | 0.25 | 0.32 | **0.000** | 0.28 | 0.25 | 0.32 | **0.000** | 0.19 | 0.16 | 0.22 | **0.000** | 0.16 | 0.13 | 0.18 | **0.000** | 0.17 | 0.14 | 0.21 | **0.000** | 0.16 | 0.11 | 0.21 | **0.000** |
| **Health status (fair)** | Ref. |  |  |  |  |  |  |  |  |  |  |  |  |  |  |  |  |  |  |  |  |  |  |  |
| Excellent | -1.06 | -1.38 | -0.74 | **0.000** | -1.29 | -1.63 | -0.95 | **0.000** | -0.85 | -1.18 | -0.52 | **0.000** | -0.74 | -1.01 | -0.48 | **0.000** | -0.98 | -1.35 | -0.60 | **0.000** | -0.91 | -1.43 | -0.39 | **0.001** |
| Good | -0.59 | -0.83 | -0.35 | **0.000** | -0.71 | -0.96 | -0.46 | **0.000** | -0.44 | -0.70 | -0.19 | **0.001** | -0.40 | -0.61 | -0.18 | **0.000** | -0.56 | -0.85 | -0.27 | **0.000** | -0.68 | -1.09 | -0.28 | **0.001** |
| Poor | 0.76 | 0.32 | 1.21 | **0.001** | 0.86 | 0.42 | 1.30 | **0.000** | 0.74 | 0.27 | 1.21 | **0.002** | 0.51 | 0.08 | 0.95 | **0.021** | 0.48 | -0.04 | 1.00 | 0.073 | 0.87 | 0.26 | 1.48 | **0.005** |
| **Continuous PCEs** | -0.40 | -0.55 | -0.25 | **0.000** | -0.30 | -0.44 | -0.15 | **0.000** | -0.24 | -0.38 | -0.09 | **0.001** | -0.34 | -0.46 | -0.22 | **0.000** | -0.30 | -0.47 | -0.13 | **0.000** | -0.33 | -0.57 | -0.09 | **0.006** |
| **PCEs (0)** | Ref. |  |  |  |  |  |  |  |  |  |  |  |  |  |  |  |  |  |  |  |  |  |  |  |
| 1 | -0.33 | -0.73 | 0.07 | 0.102 | -0.03 | -0.46 | 0.39 | 0.879 | -0.25 | -0.67 | 0.17 | 0.247 | -0.21 | -0.55 | 0.13 | 0.232 | -0.15 | -0.62 | 0.33 | 0.551 | -0.16 | -0.80 | 0.48 | 0.626 |
| 2 | -0.98 | -1.43 | -0.53 | **0.000** | -0.58 | -1.05 | -0.11 | **0.016** | -0.42 | -0.87 | 0.02 | 0.062 | -0.63 | -1.00 | -0.27 | **0.001** | -0.41 | -0.92 | 0.10 | 0.114 | -0.72 | -1.45 | 0.01 | 0.054 |
| 3 | -0.94 | -1.47 | -0.40 | **0.001** | -0.58 | -1.13 | -0.03 | **0.040** | -0.62 | -1.16 | -0.08 | **0.024** | -0.89 | -1.34 | -0.44 | **0.000** | -0.84 | -1.51 | -0.18 | **0.013** | -0.85 | -1.77 | 0.06 | 0.067 |
| 4 | -1.67 | -2.71 | -0.62 | **0.002** | -1.46 | -2.57 | -0.34 | **0.010** | -1.13 | -2.07 | -0.18 | **0.019** | -1.37 | -2.14 | -0.60 | **0.000** | -1.41 | -2.66 | -0.16 | **0.027** | -1.20 | -2.79 | 0.39 | 0.140 |
| **Neglect** | 1.38 | 1.08 | 1.69 | **0.000** | 1.14 | 0.83 | 1.46 | **0.000** | 0.85 | 0.52 | 1.17 | **0.000** | 0.89 | 0.62 | 1.17 | **0.000** | 1.04 | 0.69 | 1.40 | **0.000** | 0.98 | 0.48 | 1.47 | **0.000** |
